# Capital Growth Paths of the Neoclassical Growth Model

**DOI:** 10.1371/journal.pone.0049484

**Published:** 2012-11-20

**Authors:** Taro Takahashi

**Affiliations:** Graduate School of Agricultural and Life Sciences, The University of Tokyo, Bunkyo, Tokyo, Japan; Philipps-University Marburg, Germany

## Abstract

This paper derives the first-order approximated paths of both types of capital in the two-capital neoclassical growth model. The derived capital growth paths reveal that the short-run growth effect of capital injection differs considerably depending on which type of capital is enhanced. This result demonstrates the importance of well-targeted capital enhancement programs such as public sector projects and foreign aid.

## Introduction

For the last half century, the neoclassical model of economic growth [1] has been the most widely used starting point for analyses of long-term economic growth. Since its conception as a model with a single output produced from two inputs, capital and labour, it has been extended to cover a wide range of topics. Human capital development [2], international trade [3], technological spillover [4], cross-country differences in endowment [5], and exploitation of exhaustible resources [6] are some examples. The model has also evolved methodologically by being covered to a stochastic model [7], merged into the general equilibrium framework [8], linked to the Euler equation [9], and hybridised with the endogenous growth model [10].

These developments notwithstanding, the core of the neoclassical growth model remains its original setting. Not only is it simple and clear in concept, it is empirically testable [11], owing to the well-known reduced form equation to measure the so-called conditional convergence.

Yet despite its popularity, there has been little consensus in the literature whether the Solow model is indeed a good representation of the real economy. Some economists from the endogenous growth school believe that there is no reason to expect convergence even in the long run; and even amongst the neoclassical economists the possibility of the Malthusian trap attributable to the economy's initial condition has been raised as both a theoretical and an empirical issue [12], [13]. There is also a suggestion that the widespread test of convergence is, in fact, not a genuine test of convergence but rather a joint test of convergence and of other model specifications, given that the test result is dependent on the model's assumption regarding the technological growth [14].

Perhaps the single largest cause of this disagreement is biasedness of estimators from the convergence regression. Various types of bias has been reported in the literature: sources of upwards bias include finite sample size [15], within-group estimation [16] and the closed economy assumption commonly employed in cross-sectional analyses [17], while sources of downwards bias include omitted fixed effects [18] and inclusion of human capital into the model when in reality such capital does not significantly contribute to growth [11]. This rich collection of upwards and downwards forces makes accurate measurement of the speed of convergence challenging, with most parameters being either overestimated or underestimated. While development of empirical methodology such as use of panel data can correct the bias to a certain extent [19], there is a clear need for more theory-based solutions.

As a contribution to our understanding of the Solow model and conditional convergence on the theoretical level, this paper derives the first-order approximated paths of two types of capital when both are separately incorporated into the production function. Given the popularity enjoyed by the Solow model and growth models at large, surprisingly little attention has been paid to mechanics of capital accumulation, possibly because of difficulties involved in measurement of the capital stock. One notable exception is a series of study investigating movements of the economy's capital-output ratio [14], [20]; yet, their analyses are based on the speed of convergence equation, not the growth path of the capital-output ratio *per se*.

The capital growth paths derived in the present study have a vital policy implication in the sense that, depending on the technology possessed by the economy, injection into one type of capital has a significantly larger short-run growth effect than injection into the other type of capital; thus the former should be the injection target under publicly funded capital enhancement programs such as public sector projects and foreign aid. Equally importantly, the derived capital growth equations are empirically testable—provided that data on capital stock of the economy are available [21]. This means a potential for a new method to test conditional convergence.

## Methods

In the empirically testable version of the neoclassical growth model with two types of capital [5], [11], an economy's production at time *t* assumes a Harrod-neutral technology represented by
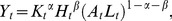
(1)where *Y* is output, *K* is the stock of physical capital, *H* is the stock of human capital, *L* is labour, and *A* is the level of technology. The Harrod-neutral technology was introduced so that when the economy-wide output is divided by effective labour *AL*, the term *A* disappears from the model to provide a simpler setting without affecting general results. The output elasticities of capital, *α* and *β*, can take any value between zero and one given that the sum of the two does not exceed the unity. The use of the aggregate production function is consistent with the view that human-capital can be interpreted as a public good [22], [23]. In addition, the assumption of the Cobb-Douglas technology has been shown to not affect the findings of the model [24].

In this model, the evolution of the two types of capital per effective labour *AL* are given, respectively, by
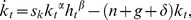
(2)and
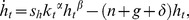
(3)where *k* and *h* are the stocks of physical and human capital per effective labour, respectively; *n* and *g* are the growth rates of population and technology, respectively; and *δ* is the depreciation rate. Following the real-life evidence [2], the depreciation rates of physical capital and human capital are assumed to be identical. The saving rates *s_k_* and *s_h_* define the proportions of the economy's output that are reinvested into physical capital and human capital. Following Equations (2) and (3), the steady-state level of the capitals per effective labour are given by
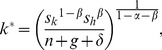
(4)and
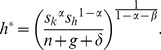
(5)Unlike the textbook case with only one type of capital [25], this model does not yield an exact growth path of the capitals because the system of *non-linear* differential equations with the form of Equations (2) and (3) does not yield analytical solutions. While it is possible to obtain numerical solutions, such an approach is not useful when qualitative properties of the system are the focus of investigation. For this reason, a first-order approximation is conducted around the steady state to analytically inspect the growth of the economy.

Now, let the steady-state values of *k* and *h* be *k* ^*^ and *h* ^*^, respectively. Then, the first-order approximations of 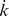 and 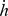 around the steady state are given by
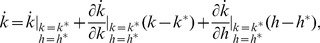
(6)and
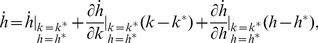
(7)where the first terms of both equations are zero. The partial derivatives of the evolution of capital with respect to capital stock can be obtained from Equations (2) and (3) as
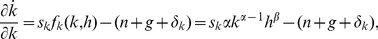
(8)

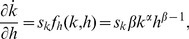
(9)

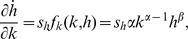
(10)and
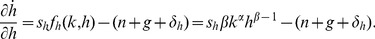
(11)Here *f_k_* and *f_h_* are the first derivatives of the production function per effective labour, *y*≡*f*(*k*,*h*) = *k^α^h^β^*, with respect to the relevant subscripts.

Finally, substituting Equations (8)–(11) into Equations (6) and (7) yields
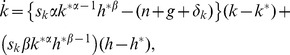
(12)and
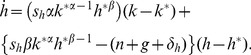
(13)Because *k* ^*^ and *h* ^*^ in Equations (12) and (13) are the steady-state values of the respective capital, they are constants. Thus, these two equations can be simplified to a system of differential equations with a general form
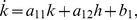
(14)and
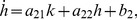
(15)where *a*_11_, *a*_12_, *a*_21_, *a*_22_, *b*_1_, *b*_2_ are all constant parameters. The solution to this system is widely known, and hence it is possible to obtain the approximate growth paths of both physical and human capital. It turns out that the solution to the system is given by
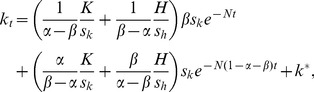
(16)and
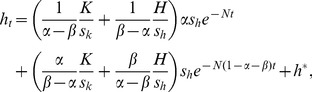
(17)where new variables *K* and *H* is defined as
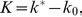
(18)and
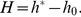
(19)
Equations (18) and (19) represent the entire distances of capital development from the initial values to the steady-state values.

The derivation of Equations (16) and (17) is given in Supporting Information S1.

## Results and Discussion

The capital growth paths derived above offer the basis to analyse the short-run effect of capital injections. The partial derivatives of Equations (16) and (17) with respect to injections *a_k_* and *a_h_* are given by
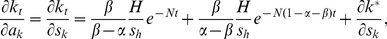
(20)

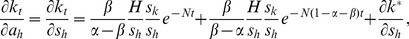
(21)

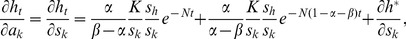
(22)and
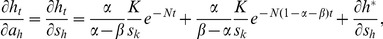
(23)where *N*≡(*n*+*g*+*δ*). The last terms of all four equations are the changes in the steady-state levels of capital attributable to the instantaneous increase in capital. Using Equations (4) and (5), they are given by
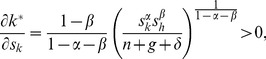
(24)

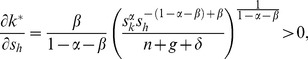
(25)

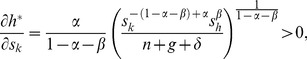
(26)and
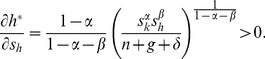
(27)The fact that all four derivatives are positive is consistent with one of the main predictions of the neoclassical models: the higher the reinvestment rates, the higher the steady-state levels of capital.

**Figure 1:**
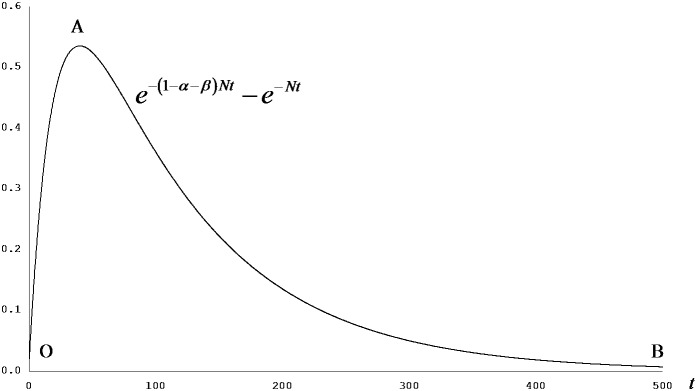
Capital growth premium: aid effect on capital stock. Equation (20) can be summarised as 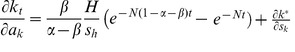, of which the terms 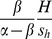 and 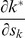 are constants. It holds that 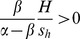 if *α>β*; from Equation (24), it also holds that 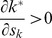 as well. Thus, once the economy has received the permanent effect of foreign aid on *k* attributable to the change in its steady-state level, 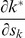, the scale of the remaining effect, hereby called the growth premium rewarding the ‘correct’ allocation of aid resource, is proportional to the term 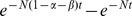 irrespective of the magnitude of the coefficient 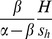. The situation is same for 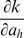 and opposite (showing the negative effect of the same absolute value) for 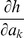 and 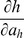. The diagram shows a sample case where *N* = 0.05 and *α*+*β* = 0.8.

**Figure 2:**
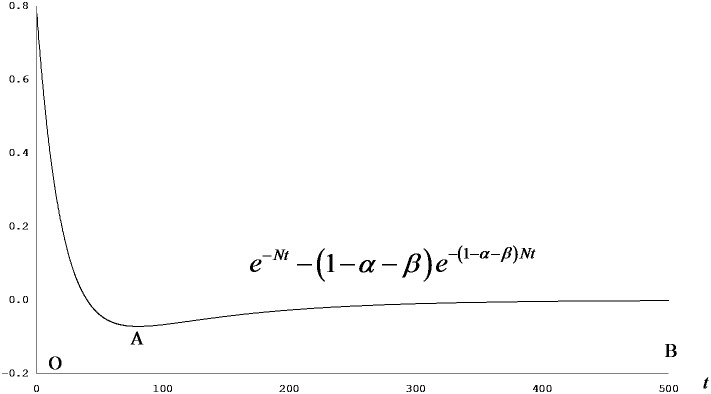
Capital growth premium: aid effect on capital formation. Equation (30) can be summarised as 
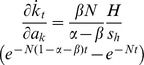
, of which the term 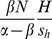 is constant and, if *α*>*β*, positive. Thus, the effect of foreign aid on the speed of capital growth is proportional to 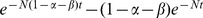 irrespective of the magnitude of the coefficient 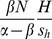. Equivalently, the curve shown in the present figure can also be derived by taking the time derivative of the curve shown in Figure 1. The situation is same for 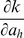 and opposite (showing the negative effect of the same absolute value) for 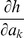 and 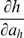. The diagram shows a sample case where *N* = 0.05 and *α*+*β* = 0.8.

The speed of capital evolution is also affected by the introduction of capital injections from outside the economy. Equations (16) and (17) yield the time derivatives of the level of physical and human capital as
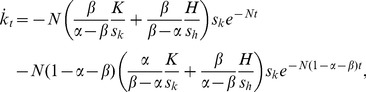
(28)and
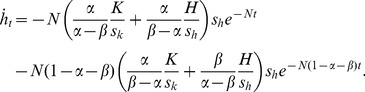
(29)Using these terms, the effects of capital injections on the speed of capital evolution are expressed as
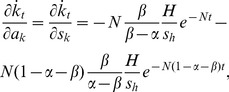
(30)

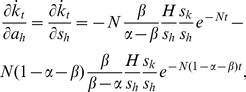
(31)

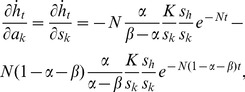
(32)and
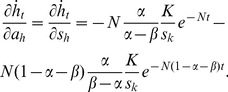
(33)
Equations (20)–(23) and (30)–(33) offer an insight about the role (or the lack thereof) of capital injection in economic growth. If the physical capital elasticity of production, *α*, is larger than the human capital elasticity of production, *β*, a one-off injection on physical infrastructure, *a_k_*, has positive growth effects on the stock levels of both physical and human capital—net of the permanent growth effect attributed to the increase in the steady-state level of capital. Conversely, an injection on social infrastructure, *a_h_*, shows negative effects on both types of capital stocks once the permanent effect has been translated into the economy. The situation is the opposite when *β>α*. Figures 1 and 2 show this point diagrammatically.

Proposition: *In the neoclassical world with two types of capital stock, capital injection from outside the economy to enhance one type of capital has a larger short-term effect than capital injection to enhance the other type of capital on the growth of both types of capital. Which type of capital shows the larger effect depends on the relative values of the capital elasticities of production*.

The existence of ‘strong’ and ‘weak’ capitals in an economy may give a partial explanation as to why the literature is divided about the effect of foreign aid on economic growth [26]. In both extremes, there are two distinct views that foreign aid unconditionally facilitates economic growth [27]–[29] and that foreign aid does not facilitate economic growth [30]–[33]. In an attempt to break the deadlock, theories have been proposed to characterise conditions under which aid facilitates growth [34], [35] although they too have been challenged [36]. Thus, it is highly inconclusive whether there exists a nexus between aid and growth [37] and there is a clear need for more theoretical work [29] as well as empirical work.

The majority of these preceding studies investigate the relationship between aid and growth by means of aid-growth regression. The finding of the present study suggests that if the country in question happens to be receiving a significant portion of the ‘wrong’ kind of aid into the weak type of capital, foreign aid will unlikely show statistical significance regardless of the amount.

In other words, aid programs that enhance the strong type of capital have strictly superior short-term effects than those that enhance the weak type of capital without compromising any long-term effect. While these temporary effects will eventually converge to zero, it is noteworthy that the purpose of foreign aid *is* to assist the recipient countries for a short term; eventual convergence and catch-up are assumed implicitly. Meanwhile, it is Pareto-efficient for donors to offer the ‘right’ kind of aid: for example more infrastructure to capital intensive economies and more training programs to labour intensive economies.

## Supporting Information

Supporting Information S1 **Proof of** **Equations (16)** **and** **(17)** **.**
(PDF)
