# Supplementary material for: Capital Growth Paths of the Neoclassical Growth Model
Source: PLoS One. 2012 Nov 20;7(11):e49484. doi: 10.1371/journal.pone.0049484 (PMC3502446; doi:10.1371/journal.pone.0049484)
Supplement: Supporting Information S1 — Proof of Equations (16) and (17) . (PDF) [file pone.0049484.s001.pdf]

**Capital growth paths of the neoclassical growth model**

**Online Supporting Information 1**

This appendix derives Equations (16) and (17) from Equations (12) and (13).

Equations (12) and (13) showed the evolution of physical and human capital by first-order approximation. They were:

$$\dot{k} = \left\{ s_k \alpha k^{*\alpha-1} h^{*\beta} - (n+g+\delta_k) \right\} (k-k^*) + \left( s_k \beta k^{*\alpha} h^{*\beta-1} \right) (h-h^*) , \quad \text{(S.1)}$$

$$\dot{h} = \left( s_h \alpha k^{*\alpha-1} h^{*\beta} \right) (k-k^*) + \left\{ s_h \beta k^{*\alpha} h^{*\beta-1} - (n+g+\delta_h) \right\} (h-h^*) . \quad \text{(S.2)}$$

Both $k^*$ and $h^*$ are the steady-state value of the corresponding capital, hence they are constants. This means that Equations (S.1) and (S.2) can be simplified into a system of two differential equations

$$\dot{k} = a_{11} k + a_{12} h + b_1 , \quad \text{(S.3)}$$

$$\dot{h} = a_{21} k + a_{22} h + b_2 , \quad \text{(S.4)}$$

where $a_{11}$, $a_{12}$, $a_{21}$, $a_{22}$, $b_1$, $b_2$ are all constant parameters. In the matrix form, the above system can be written as

$$\begin{aligned} \begin{bmatrix} \dot{k} \\ \dot{h} \end{bmatrix} &= \mathbf{A} \begin{bmatrix} k \\ h \end{bmatrix} + \mathbf{b} \\ &\equiv \begin{bmatrix} a_{11} & a_{12} \\ a_{21} & a_{22} \end{bmatrix} \begin{bmatrix} k \\ h \end{bmatrix} + \begin{bmatrix} b_1 \\ b_2 \end{bmatrix} . \end{aligned} \quad \text{(S.5)}$$

The solution to the system of linear, autonomous, first-order differential equations with form (S.5) is widely known. The system has a homogenous solution

$$\begin{bmatrix} k \\ h \end{bmatrix} = \mathbf{J}_1 e^{r_1 t} + \mathbf{J}_2 e^{r_2 t}, \tag{S.6}$$

where vector $\mathbf{J}_i$ is a solution to equation

$$[\mathbf{A} - r_i \mathbf{I}]\mathbf{J}_i = \mathbf{0}. \tag{S.7}$$

Here, $r_i$ ($i$ = 1,2) is an eigenvalue of matrix **A** and **I** is the unit matrix.

The $a_{11}$ term in Equation (S.3) is the coefficient on $k$ in Equation (S.1). It follows that

$$a_{11} = s_k \alpha k^{*\alpha-1} h^{*\beta} - (n+g+\delta). \tag{S.8}$$

Equations (4) and (5) in the main text gave the steady-state values of capitals. They were:

$$k^* = \left( \frac{s_k{}^{1-\beta} s_h{}^{\beta}}{n+g+\delta} \right)^{\frac{1}{1-\alpha-\beta}}, \tag{S.9}$$

$$h^* = \left( \frac{s_k{}^{\alpha} s_h{}^{1-\alpha}}{n+g+\delta} \right)^{\frac{1}{1-\alpha-\beta}}. \tag{S.10}$$

Substituting these into Equation (S.8) gives

$$\begin{aligned} a_{11} &= s_k \alpha \left( \frac{s_k{}^{1-\beta} s_h{}^{\beta}}{n+g+\delta} \right)^{\frac{\alpha-1}{1-\alpha-\beta}} \left( \frac{s_k{}^{\alpha} s_h{}^{1-\alpha}}{n+g+\delta} \right)^{\frac{\beta}{1-\alpha-\beta}} - (n+g+\delta) \\ &= \alpha (n+g+\delta)^{\frac{1-\alpha}{1-\alpha-\beta}} (n+g+\delta)^{-\frac{\beta}{1-\alpha-\beta}} - (n+g+\delta) \\ &= -N(1-\alpha) \end{aligned}, \tag{S.11}$$

where $N \equiv (n + g + \delta)$. Similarly, the other terms are derived as

$$\begin{aligned} a_{12} &= \beta s_k k^{*\alpha} h^{*\beta-1} \\ &= \beta s_k s_h{}^{-1} N \end{aligned}, \tag{S.13}$$

$$\begin{aligned} a_{21} &= \alpha s_h k^{*\alpha-1} h^{*\beta} \\ &= \alpha s_k{}^{-1} s_h N \end{aligned}, \tag{S.14}$$

$$\begin{aligned} a_{22} &= \beta s_h k^{*\alpha} h^{*\beta-1} - (n+g+\delta_h) \\ &= -N(1-\beta) \end{aligned} . \quad (S.15)$$

Because $0 < \alpha < 1$ and $0 < \beta < 1$, it follows that

$$a_{11} < 0, \quad (S.16)$$

$$a_{12} > 0, \quad (S.17)$$

$$a_{21} > 0, \quad (S.18)$$

$$a_{22} < 0. \quad (S.19)$$

The characteristic equation of matrix **A** is expressed as

$$r^2 - \mathrm{tr}(\mathbf{A}) + |\mathbf{A}| = 0, \quad (S.20)$$

where

$$\begin{aligned} \mathrm{tr}(\mathbf{A}) &= a_{11} + a_{22} \\ &= -N(1-\alpha) - N(1-\beta), \\ &= -N(2-\alpha-\beta) \end{aligned} \quad (S.21)$$

$$\begin{aligned} a_{11}a_{22} - a_{12}a_{21} &= |\mathbf{A}| \\ &= N^2(1-\alpha)(1-\beta) - \alpha\beta N^2 . \\ &= N^2(1-\alpha-\beta) \end{aligned} \quad (S.22)$$

Then the characteristic roots, or the eigenvalues, of the matrix is given by

$$r = \frac{-N(2-\alpha-\beta) \pm \sqrt{N^2(2-\alpha-\beta)^2 - 4N^2(1-\alpha-\beta)}}{2}. \quad (S.23)$$

Inside the square root of the right hand side of Equation (S.23) can be simplified as

$$\begin{aligned} & N^2(2-\alpha-\beta)^2 - 4N^2(1-\alpha-\beta) \\ &= N^2(\alpha^2 + 2\alpha\beta + \beta^2) \\ &= N^2(\alpha+\beta)^2 \end{aligned} . \quad (S.24)$$

Therefore, Equation (S.23) is reduced to

$$\begin{aligned} r &= \frac{-N(2-\alpha-\beta) \pm N(\alpha+\beta)}{2} \\ &= -N\frac{(2-\alpha-\beta) \mp (\alpha+\beta)}{2} \end{aligned} . \quad \text{(S.25)}$$

It follows that the distinct roots of the characteristic equations are

$$r_1 = -N(1-\alpha-\beta), \quad \text{(S.26)}$$

$$r_2 = -N. \quad \text{(S.27)}$$

Because $1 - \alpha - \beta > 0$ and $N \equiv (n + g + \delta) > 0$, the two roots have the relationship

$$0 > r_1 > r_2. \quad \text{(S.28)}$$

Extrapolating from the fact that the two roots are both negative, it is concluded that the system of differential equations (S.5) has the stable node. That is, the steady-state solutions $k^*$ and $h^*$ are asymptotically stable.

The next step is to solve the system of equations (S.7) for $r_1$ and $r_2$. The system is equivalent to

$$\begin{bmatrix} a_{11} - r_i & a_{12} \\ a_{21} & a_{22} - r_i \end{bmatrix} \mathbf{J}_i = \mathbf{0}. \quad \text{(S.29)}$$

Therefore, for $r_1 = -N(1 - \alpha - \beta)$, the eigenvector $\mathbf{J}_1$ has to be a solution for

$$\begin{aligned} &\begin{bmatrix} -N(1-\alpha)+N(1-\alpha-\beta) & \beta s_k s_h^{-1} N \\ \alpha s_k^{-1} s_h N & -N(1-\beta)+N(1-\alpha-\beta) \end{bmatrix} \mathbf{J}_1 \\ &= N \begin{bmatrix} -\beta & \beta s_k s_h^{-1} \\ \alpha s_k^{-1} s_h & -\alpha \end{bmatrix} \mathbf{J}_1 \\ &= \mathbf{0} \end{aligned} . \quad \text{(S.30)}$$

Because $N > 0$, Equation (S.30) can be simplified to

$$\begin{bmatrix} -\beta & \beta s_k s_h^{-1} \\ \alpha s_k^{-1} s_h & -\alpha \end{bmatrix} \begin{bmatrix} j_1^1 \\ j_1^2 \end{bmatrix} = \mathbf{0}. \quad \text{(S.31)}$$

This is the linear relationship between $j_1$ and $j_2$. Solving Equation (S.31) yields

$$s_k j_1^2 = s_h j_1^1. \quad \text{(S.32)}$$

Similarly, for $r_2 = -N$, the eigenvector $\mathbf{J}_2$ has to be a solution for

$$\begin{aligned} &\begin{bmatrix} -N(1-\alpha)+N & \beta s_k s_h^{-1} N \\ \alpha s_k^{-1} s_h N & -N(1-\beta)+N \end{bmatrix} \mathbf{J}_2 \\ &= N \begin{bmatrix} \alpha & \beta s_k s_h^{-1} \\ \alpha s_k^{-1} s_h & \beta \end{bmatrix} \mathbf{J}_2 \qquad . \\ &= \mathbf{0} \end{aligned} \quad \text{(S.33)}$$

Then, solving Equation (S.33) yields

$$\beta s_k j_2^2 = \alpha s_h j_2^1. \quad \text{(S.34)}$$

Finally, from Equations (S.32) and (S.34), the homogenous solution for the system (S.5) is derived. The homogenous solution is:

$$\begin{bmatrix} k_t \\ h_t \end{bmatrix} = C_1 \begin{bmatrix} s_k \\ s_h \end{bmatrix} e^{-N(1-\alpha-\beta)t} + C_2 \begin{bmatrix} \beta s_k \\ \alpha s_h \end{bmatrix} e^{-Nt}. \quad \text{(S.35)}$$

The general solution for a system of differential equations is given by the sum of its homogenous solution and its particular solution. The particular solution for the system (S.5) is the values of $k_t$ and $h_t$ when they are at the steady state. They were already given as $k^*$ and $h^*$ in Equations (S.9) and (S.10):

$$k^* = \left( \frac{s_k^{1-\beta} s_h^{\beta}}{n+g+\delta_k} \right)^{\frac{1}{1-\alpha-\beta}},$$

$$h^* = \left( \frac{s_k{}^{\alpha} s_h{}^{1-\alpha}}{n+g+\delta_h} \right)^{\frac{1}{1-\alpha-\beta}}.$$

Hence, the general solution to the system (S.5) is

$$\begin{bmatrix} k_t \\ h_t \end{bmatrix} = C_1 \begin{bmatrix} s_k \\ s_h \end{bmatrix} e^{-N(1-\alpha-\beta)t} + C_2 \begin{bmatrix} \beta s_k \\ \alpha s_h \end{bmatrix} e^{-Nt} + \begin{bmatrix} k^* \\ h^* \end{bmatrix}. \tag{S.36}$$

The remaining task is to impose the initial conditions about $k$ and $h$ on Equation (S.36) and eliminate the constant terms $C_1$ and $C_2$. Suppose that it holds at time zero that $k = k_0$ and $h = h_0$. Then, the necessary condition for Equation (S.36) to be a correct solution at time zero is

$$\begin{bmatrix} k_0 \\ h_0 \end{bmatrix} = C_1 \begin{bmatrix} s_k \\ s_h \end{bmatrix} + C_2 \begin{bmatrix} \beta s_k \\ \alpha s_h \end{bmatrix} + \begin{bmatrix} k^* \\ h^* \end{bmatrix}, \tag{S.37}$$

which can be rewritten as

$$C_1 \begin{bmatrix} s_k \\ s_h \end{bmatrix} + C_2 \begin{bmatrix} \beta s_k \\ \alpha s_h \end{bmatrix} = - \begin{bmatrix} k^* - k_0 \\ h^* - h_0 \end{bmatrix}. \tag{S.38}$$

Now, we define the distance between the initial value and the steady-state value of each capital as

$$K = k^* - k_0, \tag{S.39}$$

$$H = h^* - h_0. \tag{S.40}$$

Then, Equation (S.38) can be further simplified as

$$C_1 \begin{bmatrix} s_k \\ s_h \end{bmatrix} + C_2 \begin{bmatrix} \beta s_k \\ \alpha s_h \end{bmatrix} = \begin{bmatrix} -K \\ -H \end{bmatrix}, \tag{S.41}$$

which, again, is a system of two equations with two unknowns. Note that $K$ and $H$ are

exogenous in this model because both the initial values and the steady-state values are exogenous.

Solving the system of equations (S.41) gives the solution

$$C_1 = \frac{\alpha}{\beta-\alpha}\frac{K}{s_k} + \frac{\beta}{\alpha-\beta}\frac{H}{s_h}, \tag{S.42}$$

$$C_2 = \frac{1}{\alpha-\beta}\frac{K}{s_k} + \frac{1}{\beta-\alpha}\frac{H}{s_h}. \tag{S.43}$$

Therefore, the complete solution of the original system of differential equations (S.5) is given by

$$\begin{aligned}\begin{bmatrix} k_t \\ h_t \end{bmatrix} &= \left(\frac{\alpha}{\beta-\alpha}\frac{K}{s_k} + \frac{\beta}{\alpha-\beta}\frac{H}{s_h}\right)\begin{bmatrix} s_k \\ s_h \end{bmatrix} e^{-N(1-\alpha-\beta)t} \\ &\quad + \left(\frac{1}{\alpha-\beta}\frac{K}{s_k} + \frac{1}{\beta-\alpha}\frac{H}{s_h}\right)\begin{bmatrix} \beta s_k \\ \alpha s_h \end{bmatrix} e^{-Nt} + \begin{bmatrix} k^* \\ h^* \end{bmatrix}\end{aligned}. \tag{S.44}$$

Or separately,

$$\begin{aligned} k_t &= \left(\frac{1}{\alpha-\beta}\frac{K}{s_k} + \frac{1}{\beta-\alpha}\frac{H}{s_h}\right)\beta s_k e^{-Nt} \\ &\quad + \left(\frac{\alpha}{\beta-\alpha}\frac{K}{s_k} + \frac{\beta}{\alpha-\beta}\frac{H}{s_h}\right) s_k e^{-N(1-\alpha-\beta)t} + k^* \end{aligned}, \tag{S.45}$$

$$\begin{aligned} h_t &= \left(\frac{1}{\alpha-\beta}\frac{K}{s_k} + \frac{1}{\beta-\alpha}\frac{H}{s_h}\right)\alpha s_h e^{-Nt} \\ &\quad + \left(\frac{\alpha}{\beta-\alpha}\frac{K}{s_k} + \frac{\beta}{\alpha-\beta}\frac{H}{s_h}\right) s_h e^{-N(1-\alpha-\beta)t} + h^* \end{aligned}. \tag{S.46}$$

These are identical to Equations (16) and (17) in the main text.

From Equations (S.45) and (S.46), the optimal growth path of the income per effective

labour can also be derived. It is given by

$$\begin{aligned}
y_t &= k_t^{\alpha} h_t^{\beta} \\
&= \left\{ \left( \frac{1}{\alpha-\beta}\frac{K}{s_k} + \frac{1}{\beta-\alpha}\frac{H}{s_h} \right) \beta s_k e^{-Nt} + \left( \frac{\alpha}{\beta-\alpha}\frac{K}{s_k} + \frac{\beta}{\alpha-\beta}\frac{H}{s_h} \right) s_k e^{-N(1-\alpha-\beta)t} + k^* \right\}^{\alpha} \cdot \\
&\quad \left\{ \left( \frac{1}{\alpha-\beta}\frac{K}{s_k} + \frac{1}{\beta-\alpha}\frac{H}{s_h} \right) \alpha s_h e^{-Nt} + \left( \frac{\alpha}{\beta-\alpha}\frac{K}{s_k} + \frac{\beta}{\alpha-\beta}\frac{H}{s_h} \right) s_h e^{-N(1-\alpha-\beta)t} + h^* \right\}^{\beta}
\end{aligned} \tag{S.47}$$

Taking the logarithm of both sides of Equation (S.47) yields

$$\begin{aligned}
\ln y_t &= \alpha \ln k_t + \beta \ln h_t \\
&= \alpha \ln \left\{ \left( \frac{1}{\alpha-\beta}\frac{K}{s_k} + \frac{1}{\beta-\alpha}\frac{H}{s_h} \right) \beta s_k e^{-Nt} + \left( \frac{\alpha}{\beta-\alpha}\frac{K}{s_k} + \frac{\beta}{\alpha-\beta}\frac{H}{s_h} \right) s_k e^{-N(1-\alpha-\beta)t} + k^* \right\} . \\
&\quad + \beta \ln \left\{ \left( \frac{1}{\alpha-\beta}\frac{K}{s_k} + \frac{1}{\beta-\alpha}\frac{H}{s_h} \right) \alpha s_h e^{-Nt} + \left( \frac{\alpha}{\beta-\alpha}\frac{K}{s_k} + \frac{\beta}{\alpha-\beta}\frac{H}{s_h} \right) s_h e^{-N(1-\alpha-\beta)t} + h^* \right\}
\end{aligned} \tag{S.48}$$

Note that this is an empirically testable equation regarding the growth of national income of the country, provided that the data on the absolute level of capitals, *K* and *H*, are available.
